# Developmental Pathways Direct Pancreatic Cancer Initiation from Its Cellular Origin

**DOI:** 10.1155/2016/9298535

**Published:** 2015-11-22

**Authors:** Maximilian Reichert, Karin Blume, Alexander Kleger, Daniel Hartmann, Guido von Figura

**Affiliations:** ^1^II. Medizinische Klinik und Poliklinik, Klinikum rechts der Isar München, Technische Universität München, 81675 Munich, Germany; ^2^Division of Gastroenterology, Perelman School of Medicine, University of Pennsylvania, Philadelphia, PA 19104, USA; ^3^Department of Internal Medicine I, Ulm University, 89081 Ulm, Germany; ^4^Chirurgische Klinik und Poliklinik, Klinikum rechts der Isar München, Technische Universität München, 81675 Munich, Germany

## Abstract

Pancreatic ductal adenocarcinoma (PDA) is characterized by an extremely poor prognosis, since it is usually diagnosed at advanced stages. In order to employ tools for early detection, a better understanding of the early stages of PDA development from its main precursors, pancreatic intraepithelial neoplasia (PanIN), and intraductal papillary mucinous neoplasm (IPMN) is needed. Recent studies on murine PDA models have identified a different exocrine origin for PanINs and IPMNs. In both processes, developmental pathways direct the initiation of PDA precursors from their cellular ancestors. In this review, the current understanding of early PDA development is summarized.

## 1. Introduction

The idea that cancer cells share properties of their embryonic predecessors is, scientifically speaking, ancient [1]. Within the last decade, this conception has mostly fueled the work in the field of cancer stem cells. As controversial as this theory might be, it is one way to explain tumor heterogeneity, and solid tumors, especially pancreatic ductal adenocarcinomas (PDA), are heterogeneous [2, 3]. Similar to embryonic development, tumor cells, or at least a subset of them, have the ability to maintain indefinite growth as well as cellular plasticity. Terminal differentiation most likely has to be disadvantageous for tumor cells in order to adapt to the hostile environment within the primary tumor, the circulation, or at the metastatic site.

In the pancreas, different cell types harbor distinct susceptibilities towards oncogenic insults. Recently, new light has been shed on the cell-of-origin question of PDA. Historically PDA was thought to arise from pancreatic ductal epithelium. Instead, murine models revealed that both ductal and acinar cells are capable of transforming into distinct precursor lesions that develop into biologically different PDA subsets [4]. The parent cell transformation in both processes is characterized by dedifferentiation with recapitulation of elements of pancreatic development. Recent data investigating the role of Sox9 in PDA initiation suggest that ductal but also centroacinar cells (CACs) are more refractory to transformation mediated by a mutated* Kras* allele compared to acinar cells [5]. In contrast, Pten loss results in rapid formation of invasive carcinoma, which is preceded by significant expansion of CACs [6]. This suggests that CACs, ductal cells, and acinar cells may have the potential to initiate invasive carcinoma but that each cellular context may require a different repertoire of genetic alterations for tumour initiation. Cell-specific induction of different oncogenic mutations in mice may define morphologically and molecularly distinct tumours, which may help to identify human PDA subtypes that respond differently to therapeutic intervention.

In the following, we will give an overview of pancreas organogenesis and discuss how pancreatic cancer cells exploit developmental programs during cancer initiation with respect to their cellular origin.

## 2. Pancreatic Morphogenesis and Lineage Segregation

The development of the murine pancreas is initiated around embryonic day 8.5 (e8.5) after gastrulation, when a pancreatic and duodenal homeobox 1- (Pdx1-) expressing (Pdx1^+^) population within endodermal gut tube gives rise to both the ventral and dorsal pancreas anlage [7]. A subset of Pdx1^+^ cells that arises from the ventral foregut eventually loses its Pdx1-expression, eventually, to form the extrahepatic bile duct [8]. Stringent genetic studies in the mouse have demonstrated that Pdx1 ablation leads to pancreatic agenesis [9, 10]. The Pdx1^+^ multipotent progenitor cells (MPCs), directed by cues from the surrounding mesenchyme, establish distinct cellular lineages in order to produce and drain digestive enzymes as well as maintaining glucose homeostasis [11].

In order to execute these processes, three main lineages are required: the acinar cells, producing a plethora of digestive enzymes; the ductal cells, forming a hierarchical conduit system; and the endocrine cells, organized in the islet of Langerhans, producing hormones like insulin, glucagon, pancreatic polypeptide, somatostatin, and ghrelin. Careful designed genetic lineage-tracing studies using Cre/LoxP technology have provided insight into the spatiotemporal organization of these compartments.

For example, Gu et al. utilized* Pdx1-CreER* ^*TM*^ mice to demonstrate that the Pdx1^+^ population truly harbors multipotent progenitors since tamoxifen administration at E9.5 labels exocrine, endocrine, and duct cells [7]. Interestingly, it is the number of Pdx1^+^ progenitor cells that determines the size of the pancreas in the adult mouse, suggesting that it is rather an intrinsic program of the progenitor population than growth compensation limiting organ size, like in other organs, for example, the liver [12]. Besides Pdx1, numerous transcription factors have been employed to investigate pancreatic lineage commitment in the developing embryo. Slightly later than Pdx1 (around e9.5–10.5), Ptf1a (pancreatic transcription factor 1) is expressed in MPCs, further seizing segregation from a duodenal fate while the pancreatic bud evaginates [13]. These two transcription factors are certainly the most prominent members during this early phase of organogenesis, termed primary transition. It is at the end of primary transition, approximately around e12.5, when a primitive trunk epithelium with a continuous lumen as well as tip-structures emerges [14]. This spatial tip-trunk organization was thought to be accompanied by a loss of multipotency of the primitive duct at the beginning of secondary transition [14]. However, more recent studies, employing* Sox9Cre* ^*ER*^ as well as* Hnf1β* ^*CreERT2*^, with both transcription factors localizing to the trunk, demonstrated that either population still harbors the ability to give rise to the endocrine, acinar, and ductal lineage during secondary transition to a varying degree [15, 16].

In the adult pancreas, under tissue homeostatic conditions, Pdx1 becomes restricted to insulin-producing *β*-cells maintaining a *β*-cell-phenotype by repressing an *α*-cell program [17, 18], while Ptf1a remains expressed exclusively in acinar cells [19]. On the other hand, Sox9 and Hnf1*β* remain expressed in the ductal tree including intercalated (terminal), intralobular, and interlobular ducts as well as the main duct [20]. Thus, a set of transcription factors defines pancreatic plasticity or differentiation capacity of pancreatic progenitors in a spatiotemporally regulated manner.

The fact that mature pancreatic lineages maintain a certain degree of plasticity becomes evident in nontissue homeostatic conditions, particularly in regeneration and carcinogenesis, and will be discussed in the following section.

## 3. Pancreatic Cancer and Its Precursors

PDA is characterized by an extremely poor prognosis with a mortality rate almost equaling the incidence rate [21]. The underlying reason for this dismal situation is the limited possibility for early detection of this disease and, therefore, diagnosis is often only made in advanced stages, where only few, insufficient treatment options exist [22]. Thus, a better understanding of the initial steps of PDA development is important in order to develop new tools for early detection. In addition, deciphering the factors important for PDA progression will help to identify novel treatment options.

It is believed that PDA can develop from three established precursor lesions [23]: (i) pancreatic intraepithelial neoplasia (PanIN) and the cystic lesions, (ii) intraductal papillary mucinous neoplasm (IPMN), and (iii) mucinous cystic neoplasm (MCN). These precursor lesions differ in their prevalence; the majority of PDA is thought to arise from PanINs and less frequently from IPMN, whereas MCNs are rare [24]. There is indirect evidence for PanINs as precursors for PDA, which is largely based on the fact that PDA is often associated with advanced PanIN, and both share common tumor promoting genetic alterations. In contrast, cystic lesions can directly be identified as the origin for PDA on histological examination and imaging techniques such as MRI scan or endoscopic ultrasound. The possibility of imaging cystic precursor lesions also offers the chance to detect the precursor before PDA has developed. In fact, probably due to more frequent and better diagnostic imaging as well as physicians' awareness, IPMN lesions are increasingly identified in the pancreas, and ideal management of these patients is still an ongoing debate [25].

Interestingly, PDA that is associated with IPMNs has a much more favorable prognosis than PDA that is thought to arise from PanINs [26–28]. The underlying reasons for this different biological behavior are largely unknown. One possibility could be different genetic mutations during evolution of PDA from its precursors. In fact, whereas a* KRAS* mutation occurs nearly universally during PanIN initiation [29],* KRAS* is less frequently mutated in IPMNs [30, 31]. Instead, IPMNs but not PanINs frequently harbor mutations in* GNAS* and* RNF43* [32–34]. Apart from this difference, common genetic alterations in both precursors are found in* TP53* and* CDKN2A* (reviewed by Xiao [35] and Gnoni et al. [24]).

An additional possibility for the different biology of PanIN and IPMN-associated PDA could be a different cellular origin of the precursors. In line with this hypothesis, recent evidence from genetically engineered mouse models (GEMM) revealed that the cellular origin of PanINs and IPMNs might be different [4, 5]. This work will be discussed below.

## 4. Mechanisms of PanIN Development from Its Cellular Origin

Whereas there is considerable knowledge about the molecular and genetic events during progression of PanIN lesions, the mechanisms of precursor initiation are still poorly understood. Historically, PDA and its precursors were thought to develop from pancreatic ductal cells because both have a ductal morphology and express ductal markers such as cytokeratin 19 (CK19). However, this assumption was challenged by studies in mice that revealed an acinar source for PanIN lesions [36–38].

### 4.1. ADM/ADR: The Precursor of the Precursor?

That pancreatic acinar cells have a marked plasticity was already noted 30 years ago in pancreatitis studies on rats [39, 40]. In these experiments an acute pancreatitis was induced by repetitive injections of cerulein, a cholecystokinin analogue, which causes autodigestion of the pancreas and a pronounced inflammatory reaction. It was found that in response to this damage acinar cells form a transient duct-like metaplasia before a complete regeneration of the organ occurs. The direct* in vivo* evidence for an acinar source of this acinar to ductal metaplasia (ADM) was brought in 2008 using lineage-tracing techniques on a murine pancreatitis model by Fendrich et al. [41]. Further characterization of ADM has shown that it is not only accompanied by downregulation of acinar and expression of ductal markers (e.g., CK19) but also resembles pancreatic embryonic progenitor cells evidenced by reexpression of pancreatic developmental factors, such as PDX1, Sox9, and Hes1 [41–43]. Following this transient phase in response to an acute damage, the duct-like cells of ADM resume an acinar morphology and expression profile [43, 44]. In contrast to an acute damage, chronic pancreatitis leads to a persistence of ADM [45] and a failure to regenerate the pancreas. However, although acute and chronic pancreatitis can induce duct-like structures originating from pancreatic acinar cells, both are not sufficient to induce PanIN lesions* per se*.

Importantly, an oncogenic mutation in* Kras* can induce ADM from acinar cells that resembles ADM formed in response to pancreatitis [37, 46]. However, the Kras-induced ADM is persistent and not transient and is also termed acinar to ductal reprogramming (ADR) in this context. In addition to ADR, acinar expression of mutant Kras is sufficient to induce PanIN lesions [36–38, 46]. Although direct lineage-tracing evidence is missing, it is suggested that Kras-associated ADR progresses to PanIN lesions [47], in part, because the expression of key signaling pathways in ADR cells is mimicking expression detected in PanIN lesions [48]. Moreover, in mouse models with expression of mutant Kras in the pancreas lesions of acinar to ductal reprogramming precede PanIN development [43, 48]. In addition, PanINs in mice and humans are usually associated with areas of ADM/ADR [48].

### 4.2. Acinar Cells Give Rise to PanINs

In contrast to mouse models where mutant Kras is activated during pancreatic embryogenesis [49], mutant Kras expressed in mature acinar cells leads to PanIN formation but is insufficient to cause PDA [36–38, 46]. However, when combined with chronic pancreatitis, which is a risk factor for the development of PDA [50], mutant Kras (*Kras* ^*G12V*^) was able to accelerate precursor formation with progression to cancer [37]. This suggests that inflammation can induce synergistic protumorigenic changes in acinar cells that cooperate with mutant Kras. Pancreatitis experiments in wild-type animals have shown that these changes could comprise reactivation of developmental factors that have been shown to be important for Kras-mediated neoplastic transformation of pancreatic acinar cells which will be discussed in detail below [42, 43].

**Figure 1:**
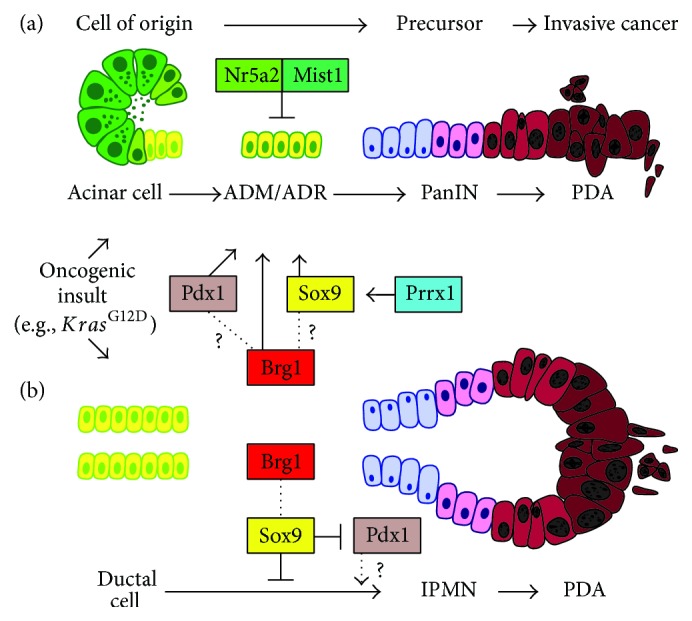
A model for developmental factors regulating acinar and duct cell transformation. (a) Acinar cells undergo acinar to ductal reprogramming (ADM/ADR) on their way to become PanINs and PDA. The ductal reprogramming of acinar cells is inhibited by factors that maintain stable acinar differentiation, such as Mist1 and Nr5a2. Instead, the transcription factors Sox9 and Pdx1 promote ductal reprogramming. Prrx1 and the chromatin remodeler Brg1 also promote ductal reprogramming, possibly by regulating other transcription factors, such as Sox9 or Pdx1. (b) Ductal cells transform into IPMN lesions by undergoing a dedifferentiation step (“ductal retrogression”) evidenced by an upregulation of the progenitor marker Pdx1. The chromatin remodeler Brg1 suppresses ductal retrogression by regulating Sox9 expression, which in turn inhibits Pdx1. This points to an opposing function of Brg1 in acinar versus duct cell transformation.

The above mentioned studies have shown that adult pancreatic acinar cells are capable of forming PanIN lesions in the context of mutant Kras. It is also important to note that ductal cells in CK19 promoter-based expression of mutant Kras were able, albeit at much lower frequency, to form PanIN lesions [51]. In a study by Kopp et al. it was investigated by comparative recombination in adult pancreatic duct/CACs and acinar cells using tamoxifen inducible* Sox9CreER* and* Ptf1a* ^*CreER*^-mediated recombination of mutant Kras [5]. Interestingly, it was found that pancreatic ductal cells including CACs were virtually incapable of transforming into PanIN lesions, whereas acinar cells readily transformed into PanIN lesions with a >100-fold greater efficiency as compared to ductal cells. Scientists in the field have speculated extensively about the possibility of CACs being the cell of origin of PanIN and PDAC. However, the compartment of CACs is still characterized insufficiently and, hence, the genetic tools to address this question appropriately are missing. Taken together, the most recent findings suggest that this is a very unlikely scenario and strongly support the model that at least in the murine Kras model acinar cells serve as the origin for PanIN lesions. This also prompts the question of which factors are important for acinar cells to transform into ADR/PanIN. Previous studies have taught us that recapitulation of developmental factors is occurring during acinar transformation. One of these is the embryonic transcription factor Sox9 that is expressed in pancreatic progenitor cells and becomes restricted to ductal cells in the adult organ [52]. Kopp et al. investigated if this factor not only is a marker of acinar cells undergoing transformation but also plays an essential function in this process [5]. To test this, Sox9 was deleted from acinar cells expressing oncogenic Kras, which resulted in a complete blockage of PanIN formation (Figure 1). Vice versa, overexpression of Sox9 in the context of oncogenic Kras dramatically catalyzed preneoplastic transformation. Mechanistically, it was shown that ectopic expression of Sox9 alone in acinar cells can erode cell integrity evidenced by downregulation of markers of acinar differentiation, such as Mist1, and concomitant upregulation of the ductal factor CK19 [5]. Interestingly, previous studies have shown that combining mutant Kras with a deletion of Mist1 also accelerates formation of PanIN lesions in the pancreas [53], which further highlights the relevance of a stable acinar differentiation state as a barrier for Kras-mediated transformation (Figure 1).

### 4.3. Epithelial Cell Plasticity

The notion that loss of acinar cell integrity is a prerequisite for PDA formation is accompanied by the emergence of a developmental program as mentioned above [44]. This phenomenon is not exclusive to pancreatic cancer but also present in other processes involving ADM during pancreatic regeneration in response to pancreatitis [42]. Besides the already mentioned Sox9 and Pdx1, Notch- and Shh-signaling, among others, are reactivated within the acinar compartment after challenging the gland using cerulein [42]. Interestingly, persistent overexpression of Pdx1 within the pancreatic epithelium by transgenic means leads to ADM formation suggesting that Pdx1 has a driving function in acinar cell dedifferentiation [54] (Figure 1). More recently, using an unbiased approach comparing gene expression profiles of the developing pancreas, acute pancreatitis and mutant* Kras*-driven carcinogenesis revealed a transcriptional program shared in these processes [55]. Of note, Prrx1 emerged as the most regulated transcription factor in this system. The* Prrx1* gene encodes two major variants,* Prrx1a* and* Prrx1b*, generated by alternative splicing [56]. Remarkably, PRRX1B is upregulated in ADM as well as PanINs and induces Sox9 expression on a transcriptional level thereby fostering a ductal phenotype [55] (Figure 1). In PDA, both splice variants, Prrx1a and Prrx1b, control differentially epithelial-to-mesenchymal transition (EMT) and the reverse process, mesenchymal-to-epithelial transition (MET) (Takano, Reichert et al. unpublished data). In general, the degree of cellular plasticity is illustrated by the ability to undergo EMT/MET both being critical in embryonic development (EMT type I), tissue regeneration (EMT type II), and cancer (EMT type III) [57]. The two latter types are characterized by an inflammatory response in contrast to EMT type I observed in the embryo [58]. However, all subtypes can be identified by the expression of EMT-transcription factors (EMT-TFs), including Slug, Snail, Twist, and Zeb [59]. Interestingly, Slug has been found to cooperate with Sox9 in promoting tumorigenicity in breast cancer cells [60].

### 4.4. Acinar Cells in Human Pancreatic Cancer Initiation

In summary, murine studies have identified acinar cells as the origin for PanIN lesions and highlighted the role of developmental factors in this process. An open question remains if these studies in mouse models are applicable to the situation of pancreatic cancer initiation in humans. Although it is impossible to directly prove* in vivo *that acinar cells transform into PanINs in human pancreas, there is some evidence that this process may also be true for human pancreatic carcinogenesis.* In vitro* studies have shown that human pancreatic acinar cells have a comparable plasticity, as their murine equivalents, and readily transdifferentiate into duct-like cells in culture [61]. Shi et al. examined in human pancreas specimens if the initiating oncogenic Kras mutation can be detected in acinar cells that are in proximity to ADM/PanIN [62]. Although the authors could not detect Kras mutations in acinar cells and isolated areas of ADM, expectedly PanINs but also areas of adjacent ADM exhibited a high frequency of mutant Kras. This data could be interpreted that ADM adjacent to PanIN represents retrograde extensions of the latter and thus would not support an acinar origin for PanINs. However, it is also conceivable that it was impossible to detect mutant Kras in morphologically normal acinar cells, because Kras mutant acinar cells may promptly transdifferentiate into ADM in humans and Kras mutant ADM may have a high propensity to immediate formation of PanIN lesions.

The acinar origin hypothesis is also supported by genome wide association studies that identified frequent single-nucleotide polymorphisms (SNPs) in pancreatic developmental factors that are associated with pancreatic cancer. These developmental factors comprised not only* PDX1* but also the important regulators of acinar differentiation* HNF1A* and* NR5A2* [63, 64]. The role of Nr5a2 in maintaining a stable acinar differentiation state and preventing Kras-driven PanIN formation was subsequently demonstrated in studies in mice [65, 66] (Figure 1). These investigations supported the relevance of the identified SNPs in* NR5A2* as pancreatic cancer susceptibility lesions and further suggest a role of acinar cells in human preneoplastic transformation.

## 5. Development of IPMN from Its Cellular Origin

Whereas PanINs and PanIN-PDA have been studied in great detail, in part, due to the prevalence of GEMM recapitulating PanIN-PDA formation [67, 68], the molecular properties of IPMN are less well characterized and few suitable mouse models for studying IPMN-PDA progression had been developed [69, 70]. In humans, IPMNs are macroscopically cystic lesions that develop within the pancreatic main and/or branch duct system and show a direct connection to the ducts. This fact and the ductal morphology have led to the assumption of duct cells as the cellular origin for this precursor lesion. However, lineage-tracing studies in murine models had been hampered by the lack of suitable inducible* Cre*-lines that allow recombination specifically in adult duct cells. Therefore, proving a ductal origin of the preneoplastic lesions in mouse models of IPMN had been impossible.

With the recent availability of such duct-specific* Cre*-lines, it was demonstrated in a study on a novel GEMM of IPMN that the preneoplastic lesions in this particular model derive from ductal cells [4]. In an initial experiment, it was shown that embryonic pancreas specific deletion (*Ptf1a* ^*Cre*^) of Brg1, the catalytic subunit of the SWItch/sucrose nonfermentable (SWI/SNF) chromatin remodeling complexes, in combination with expression of mutant Kras led to the formation of cystic neoplastic lesions in adult mice that resembled human IPMNs [4]. This mouse model faithfully recapitulated human IPMN-PDA development and mirrored the human situation with a less malignant biological behavior as compared with PanIN-PDA. Next, it was interrogated, in this model, whether the IPMN lesions were of ductal or acinar origin by using* Cre*-lines that allow specific recombination of the* Brg1* and mutant* Kras* alleles in adult acinar (*Ptf1a* ^*CreER*^) or ductal (*Hnf1β-Cre* ^*ERT2*^) cells [4]. These experiments showed that Brg1 deletion renders adult duct cells sensitive to Kras-mediated transformation evidenced by the occurrence of duct atypia and lesions that resembled IPMNs. In contrast, Brg1 deletion in adult acinar cells in the context of mutant* Kras* did not lead to IPMN formation and, moreover, PanIN formation was blocked. This suggests that Brg1 plays a dual role in PDA precursor development by inhibiting duct transformation to IPMN and promoting acinar transformation to PanIN in the context of an oncogenic stimulus by mutant* Kras *(Figure 1).

This study gave the first experimental evidence that ductal cells can serve as the origin for IPMN lesions and highlighted the relevance of chromatin remodeling in this process. In a subsequent work, Roy et al. investigated the mechanistic basis for this observation [71]. It was found that combination of mutant* Kras* and loss of Brg1 in pancreatic ducts led to a dedifferentiation of mature ductal cells that was termed “ductal retrogression.” Ductal retrogression was characterized by a reduced expression of markers of mature ductal differentiation and an upregulation of pancreatic progenitor factors such as Pdx1. One of the downregulated ductal markers was Sox9. Mechanistically it was shown that ectopic expression of Sox9 in the context of combined mutant Kras/Brg1 loss prevented ductal retrogression and IPMN formation (Figure 1). The upregulation of Pdx1 during ductal retrogression mirrors the expression pattern in pancreatic acinar cells during injury or Kras-mediated dedifferentiation. This points to Pdx1 as a unique factor in transformation of both acinar and ductal cells. Therefore, future studies need to explore a functional role of Pdx1 in this process and whether inhibition of this progenitor factor can prevent the formation of both PanIN and IPMN.

## 6. Conclusions with Translational Aspects

In contrast to some other cancers, embryonic signaling pathways like TGF*β*, Wnt-*β-*catenin, and Hedgehog alone are not sufficient for the initiation of PDA [44]. Although the expression of a primitive ductal program can be launched, an oncogenic insult, most frequently mutated and constitutive active Kras, is required to drive pancreatic cancer progression. Apparently, acinar cells display the highest degree of cellular plasticity in order to adopt an undifferentiated progenitor state upon inflammatory or oncogenic stimuli. At the same time, it is becoming more and more clear that, although morphologically relatively uniform, PDA represents an extremely complex disease. In the field of pancreatic cancer research, we are lagging behind in terms of subtype identification compared to other solid cancer entities, for example, breast cancer. The cell of origin or precursor type leading to PDA has significant impact towards the prognosis of PDA patients. This suggests that not only the genetic makeup of a given cancer cell but its primordial lineage plays an important role. Our knowledge has been fueled to a large extent by mouse models of pancreatic cancer but in order to address the complexity of this disease, human model systems are needed. For example, Kim et al. used an elegant induced-pluripotency approach to reprogram human PDA cells in order to recapitulate human disease progression [72]. Another study by Boj et al. suggests that an organoidculture system established from surgery specimen or endoscopic biopsy material grown in a three-dimensional matrix might be a useful tool to address this complexity [73]. In addition, this model system harbors the opportunity to test personalized therapies for pancreatic cancer patients, potentially even in real time.

In general, mouse models have significantly contributed to our understanding of virtually all aspects of PDA biology. However, so far we were not able to make use of this knowledge in order to improve PDA patients' care. The recent advances in pancreatic cancer treatment have been generated by not molecular defined strategies but rather pragmatic approaches using extremely toxic chemotherapeutic regimens [74] or increasing the delivery of established cytotoxic drugs [75].

The genetic heterogeneity of PDA and distinct oncogenic susceptibilities of defined compartments within the gland make this disease the opposite of what clinicians call a “chameleon”; one disease with many faces, PDA, instead, might represent numerous diseases with the same appearance. Thus, tailored therapies taking the mutational landscape of the respective tumor into account need to be developed and an even more profound understanding of the cellular plasticity and the regulating genetic factors in pancreatic carcinogenesis is required.
